# Patients with ST segment elevation myocardial infarction: moderating effect of perceived control on the relationship between depression and in-hospital complications

**DOI:** 10.1186/s12872-019-1126-z

**Published:** 2019-06-13

**Authors:** Mohannad Eid AbuRuz

**Affiliations:** Clinical Nursing Department, Faculty of Nursing, Applied Science Private University, Po Box 142 Shafa Badarn, postal code, Amman, 11934 Jordan

**Keywords:** Acute myocardial infarction, Depression, Perceived control, Complications

## Abstract

**Background:**

Cardiovascular diseases remain the top global killer, with nearly 80% of related mortalities occurring in developing countries. Over half of cardiovascular diseases’ mortality is due to coronary heart disease, which is commonly linked to acute myocardial infarction. Psychological factors (i.e., depression and anxiety) after acute myocardial infarction are associated with higher levels of complications and mortality. Perceived control moderated the effect of anxiety on complications in different cardiac populations, but impacts on depression and complications after acute myocardial infarction are not well studied. This study explores the moderating effect of perceived control on the relationship between depression and complications after ST segment elevation myocardial infarction.

**Methods:**

Three hundred patients with a confirmed diagnosis of ST segment elevation myocardial infarction participated in this prospective observational study. Patients answered socio-demographic data, the depression subscale of the Hospital Anxiety and Depression Scale (HADS), and the Control Attitude Scale-Revised (CAS-R) questionnaires. In-hospital complications and all other necessary data were extracted from medical records after discharge. Data were analyzed using logistic regression.

**Results:**

24% developed at least one complication. Patients with high depression scores (8–21) were more likely to develop complications (χ^2^ = 34.15, *p* < .001) than those with low depression scores (0–7). Patients with high levels of perceived control had lower levels of depression than those with low perceived control (mean [SD], 9.47 [6.43] vs. 12.31 [6.66], *p* < .001). The results of logistic regression showed that perceived control moderated the association between depression and complications, since depression scores, perceived control scores, and the interaction between depression and perceived control were significant predictors of complications. Participants with high depression and low perceived control had the highest rate of complications (31.5% vs. 15.4%, *P* < .001).

**Conclusions:**

Depression increased complications after ST segment elevation myocardial infarction. Perceived control moderated this relationship. Assessment of depression and enhancement of perceived control in patients with acute myocardial infarction can decrease complications and improve outcomes.

## Background

Cardiovascular diseases (CVDs) are the most common cause of death worldwide [1]. In the US alone, there is more than 90 million diagnosed with at least one type of CVD [1]. Approximately 80% of all deaths nationwide due to CVDs occur in developing (low and middle income) countries [1]. In the developing countries of the Middle East, mortality rates due to CVDs are increasing [2]; in Jordan, 35% of all deaths occur as a result of CVDs [3].

More than half of all CVDs are due to coronary heart disease (CHD) [1]. Every 40 s an American will have an acute myocardial infarction (AMI) due to CHD. In 2019, it is estimated that 720,000 Americans will have a new AMI, and 335,000 will have a recurrent event [1]. Nearly 35% of patients who experience a CHD event yearly will die from it, and ≈15% who develop AMI will die of it [1]. In Jordan, 131 deaths per 100,000 are due to CHD, accounting for nearly 20% of all deaths [3].

In the US, during the period from 2001 and 2011, in-hospital mortality after ST segment elevation myocardial infarction (STEMI) increased for patients without any intervention, did not change for patients who received percutanous coronary intervention, and decreased for patients who had coronary artery bypass surgery [1]. Therefore, determining physiological and psychological factors affecting mortality and morbidity for patients with STEMI is warranted.

Psychological manifestations after AMI are very common, with the most common being depression and anxiety. The incidence of depression after AMI might be as high as 80% [4, 5]. Previous studies have found evidence that depression is associated with short- and long-term complications after AMI. In the short term, starting as early as the first 20 min after AMI [6–9], depression was an independent predictor of complications such as acute recurrent ischemia, re-infarction, ventricular tachycardia, ventricular fibrillation, cardiogenic shock, pulmonary edema, inflammation (i.e., endocarditis), left ventricular mural thrombus, and in-hospital death [3, 4, 6, 7, 10]. Moreover, high levels of depression were associated with higher levels of fatigue and longer hospitalization, especially in critical care units [4], and lower levels of left ventricular ejection fraction (LVEF) [11].

Over the long term, depression is a more significant predictor than traditional risk factors such as smoking and hyperlipidemia for adverse outcomes after AMI [12, 13]. Depression was associated with increased risk of re-infarction, readmissions [14, 15], and ischemic cardiac events [16–18]. Moreover, in longitudinal studies (up to 10 years post-event) depression increased morbidity and mortality after AMI [19, 20]. In addition, depression played a significant role of incomplete recovery [21], poor quality of life [22, 23], postponing return to work [24], lack of adherence to medication and health care team instructions [25], and not following the rehabilitation protocols after AMI [26].

The effect of depression on cardiac mortality after AMI was assessed in a scientific statement from the American Heart Association based on their analysis of 11 studies. A significant relationship was reported in 8 studies [27]. Based on the results of their analysis, the American Heart Association reinforced their appraisal of depression as a risk factor for complications and mortality after AMI [27]. Patients’ coping, psychosocial repossession, and quality of life after AMI depends on psychological (i.e., depression) instead of physiological factors [3, 28]. It has been shown that personal control and social support have protective effects against depression, and improve quality of life in different cardiac populations [29, 30]. Therefore, if depression is assessed and managed well for patients with AMI, this might improve their outcomes and decrease complications.

Perceived control (PC) is a new strategy under investigation that might have a protective effect against depression in patients with AMI. It has been defined as “an individual’s belief that he or she has the resources required to cope with negative events in a way that positively influences their adversive nature” [28]. To our knowledge, no studies were specifically designed to check the effect of PC on depression and in-hospital complications in patients with AMI. However, different studies investigated the relationship between PC and anxiety, finding that PC was negatively associated with anxiety in AMI, cardiac surgery, and heart failure [28]; moreover, it was an independent predictor of anxiety in these populations, and moderated the relationship between anxiety and in-hospital complications [3, 31]. Therefore, the major purpose of this study was to check if there is a moderating effect of PC on the relationship between depression and in-hospital complications after STEMI.

## Methods

### Research design, sample, and setting

This study employed a prospective, non-experimental, observational design, recruiting participants from one governmental and two private hospitals in Amman. Inclusion criteria comprised: (1) cardiology STEMI diagnosis (confirmed), with increased enzyme and cardiac changes (indicated by ECG); (2) adult patients (aged over 18 years); (3) not in unusual pain and stable hemodynamically at the time of interview; (4) able to give (and sign) informed consent and to participate by answering questionnaire items; and (5) not suffering from critical comorbidities and serious non-cardiac conditions (e.g. stroke, sepsis, and shock). Exclusion criteria included those who prior to PC or depression manifested in-hospital complications, in order to explore the longitudinal, cause-and-effect dimension of the clinical problem.

**Fig. 1:**
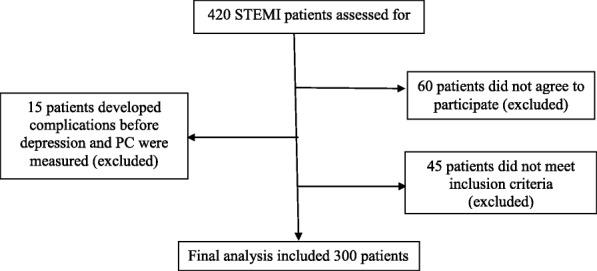
Patient flow diagram. A total of 300 patients were included in the final analyses PC: Perceived control.

A logistic regression sample-size calculator was used a priori to ensure that the findings would have significance statistically. Criteria included estimated event rate (occurrence of complications) of 27% [31], two-tailed test with power of 0.8, and alpha of 0.05. Regression analysis was conducted with 13 independent variables. The consequent requirement was 238 participants, thus 300 participants were included to account for dropout and attrition etc. (Fig. 1).

### Ethical considerations

The Applied Science Private University Amman’s Institutional Review Board (IRB) Committee analyzed this study and accorded it their ethical approval (IRB#: Faculty 024). Consequently, the University President formally requested that the hospital directors enable and assist the fieldwork. Subsequently, the medical directors in the studied settings were met by the principal investigator to ensure their acknowledgement and cooperation with the approval letter, and they issued their own permission to begin data collection and to apply the study fieldwork.

### Procedure

Research assistants (RAs) were engaged to collect data from hospitals, with critical care nursing master’s degrees, and training in cardiovascular care. Each potential participant in the studied settings who met the inclusion criteria was approached by the RAs, who explained the study to them in depth, along with all pertinent ethical information (e.g. voluntary participation and right to withdraw etc.), and those who subsequently wished to take part were asked to sign a form indicating their informed consent. Participants were interviewed while hemodynamically stable during their initial 3 days following admission (mean ± SD; 35 ± 16 h). Socio-demographic information was recorded by the RAs, who also delivered the Control Attitude Scale-Revised (CAS-R) and the depression subscale of the Hospital Anxiety and Depression Scale (HADS). Upon discharge, RAs noted from participants’ medical records their in-hospital complications (if applicable) along with any other pertinent comorbidities.

### Measurement of variables

- Clinical and Socio-Demographic Characteristics

As mentioned previously, following participants’ discharge from hospital (i.e. upon completion of their in-patient treatment), the RAs extracted socio-demographic and clinical data from their medical records, including gender, age, smoking profile, vital signs upon admission and chest pain severity, and experience of diabetes mellitus, emergency and ICU medications, hypertension, LVEF, and myocardial infarction.

- Depression

Arabic HADS was used to assess depression. Numerous cardiac studies affirm its psychometric proprieties (Cronbach’s α 0.87) [6, 32–35]. The seven items are rated on a four-point Likert scale, ranging from 0 to 3, where 3 refers to the maximum severity and frequency of symptoms. The values assigned for each item add up to a net score ranging from 0 to 21, which are then classified according to depression severity as normal (0–7), mild (8–10), moderate (11–14), and severe (15–21) [6, 32].

- In-Hospital Complications

STEMI frequently results in in-hospital complications [3, 4, 7, 8, 31], of which the following are common: (a) ventricular fibrillation; (b) ventricular tachycardia warranting care, particularly ≥30 s (attributable to hemodynamic instability); (c) acute recurrent ischemia; (d) cardiogenic shock; (e) reinfarction; (f) acute pulmonary edema; and (g) mortality.

- Perceived Control

Arabic CAS-R was deployed to measure PC, having displayed acceptable psychometric proprieties among cardiac populations. Hypothesis testing has displayed its construct validity for known associations, and its reliability and validity have been demonstrated among 500 STEMI participants (Cronbach’s α 0.85) [3]. The tool comprises eight items answerable with Likert-type scales, with responses descending from 5 “totally agree” to 1 “totally disagree”. Net scores are in the range of 8–40, with lower scores denoting lower PC levels. Due to the lack of mean norms, other researchers deployed median values as delimitation points [3, 31]; following this method, the resultant median in this study was set as the cut-off point (29), whereby participants with higher scores for PC had high PC, and vice-versa.

### Data analysis

Data analysis was conducted with recourse to SPSS (version 24). Statistical significance was denoted by *p* value < .05. Clinical and socio-demographic characteristics related to depression changes at baseline were described by frequencies, percentages, and Mean ± SD. Continuous and categorical variables were analyzed using Student’s t-test or χ^2^, respectively, to ensure that the studied variables were pertinent to the research. Variables differing between the high and low depression groups could thus be controlled for the purposes of subsequent analyses.

To control for the other variables and to determine the impacts of PC, depression, and interaction term (moderating effect), multiple hierarchal logistic regression was deployed, yielding results as odds ratios and 95% confidence intervals. Three blocks were applied for regression: (1) age and gender; (2) history of diabetes, emergency department medication (e.g. beta block, aspirin, or anti-depressant), hypertension, LVEF, previous myocardial infarction, and smoking; and (3) PC and depression scores, and depression*PC interaction. Multicollinearity between variables was absent, as indicated by variance inflation factor (less than 3).

## Results

### Socio-demographic and clinical characteristics

The sample consisted of a total 300 participants, including 231 men and 69 women. During hospitalization, 72 participants (24%) developed at least one complication (Table 1). Patients with high depression scores (8–21) were more likely to develop complications (χ^2^ = 34.15, *p* < .001) than those with low depression scores (0–7). Moreover, they have lower levels of LVEF (M [SD], 44.60 [7.31] vs. 53.05 [7.12], p < .001). Patients with high levels of PC had lower levels of depression than those with low PC (M [SD], 9.47 [6.43] vs. 12.31 [6.66], p < .001). Socio-demographic and clinical characteristics relative to depression levels are presented in Table 2. Only one clinical variable differed between low and high depression groups: patients in the high depression group received anti-depressant medication more frequently than those in the low depression group Table 3. There were no differences in any socio-demographic or clinical variables between those who continued the study and those who dropped out.

**Table 1:** Specific complications developed and their percentages

Complication developed	^a^Number of patients (%)
Acute recurrent ischemia	30 (41.7)
Pulmonary edema	10 (13.9)
Sustained ventricular tachycardia	9 (12.5)
Re-infarction	8 (11.1)
Cardiogenic shock	7 (9.7)
In-hospital death.	3 (4.2)
Ventricular fibrillation	2 (2.8)

^a^More than one patient developed more than one complication

**Table 2:** Sociodemographic and clinical characteristics of the sample based on depression levels (*N* = 300)

Characteristic	High Depression (*n* = 195)	Low Depression (*n* = 105)
Age	69.5 ± 9.0	69.02 ± 10.0
Gender		
Male	149 (76.4)	82 (78.1)
Female	46 (23.6)	23 (21.9)
History of DM	80 (41.0)	49 (46.7)
History of HTN	155 (79.5)	86 (81.9)
History of previous AMI	122 (62.6)	75 (71.4)
History of smoking	146 (74.9)	83 (79.0)
Severity of chest pain	5.17 ± 2.2	5.26 ± 2.2
Ejection fraction	44.6 ± 7.3	53.05 ± 7.1**
Development of complications	59 (30.3)	48 (18.8)**

Values are presented as M ± SD or n (%), *DM* Diabetes Miletus, *HTN* Hypertension, *AMI* Acute myocardial infarction, ** significant at *P* < .001

**Table 3:** Treatment received during hospitalization (N = 300)

Treatment	High Depression (n = 195)	Low Depression (n = 105)
Thrombolytic agents	80 (41.0)	37 (35.2)
Beta blocker	98 (50.3)	50 (47.6)
Aspirin	175 (89.7)	90 (85.7)
Anti-depressant	113 (58.0)	42 (40.0)*
Coronary artery bypass graft	20 (10.3)	12 (11.4)
Angioplasty	119 (61.0)	67 (63.8)

Values are presented as n (%).* significant at *P* < .05

### Checking the moderating effect

**Fig. 2:**
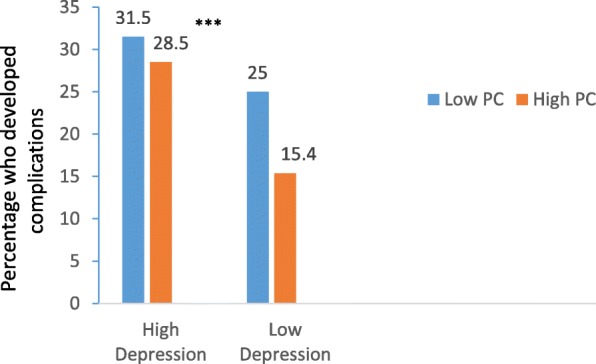
Comparison of percentage of patients who developed complications based on depression levels and PC. Patients with high depression and low PC had the highest complication rate indicating the moderating level of PC on the relationship between depression and complications. ***p < .001. Abbreviation: PC, perceived control.

The results of the logistic regression are presented in Table 4. The model shows five significant predictors: history of previous AMI, depression scores, PC scores, LVEF, and the interaction between depression and PC. Perceived control moderated the association between depression and complications since depression scores, PC scores, and the interaction between depression and PC were significant predictors of complications. Participants with high depression and low PC have the highest rate of complications (31.5% vs. 15.4%, *p* < .001) (Fig. 2).

**Table 4:** Logistic regression analysis for predictors of in-hospital complications

Predictor	Odds ratio	Wald	95% CI	p value
History of previous AMI	2.21	8.61	1.51–4.31	.005
Depression scores	1.51	7.70	1.11–2.01	.007
Perceived control	0.81	7.01	0.75–0.99	.008
LVEF	0.83	6.22	0.71–0.96	.030
Depression scores * Perceived control	1.61	8.31	1.22–2.21	.006

*AMI* Acute myocardial infarction, *CI* Confidence interval, *LVEF* Left ventricular ejection fraction. Variables used in the model (age, gender, history of diabetes, history of hypertension, history of smoking, history of previous AMI,, beta blocker use, anti-depressant use, aspirin use, left ventricular ejection fraction, depression scores, perceived control scores, and the interaction between depression and perceived control)

### Other important findings

Previous myocardial infarction and high levels of depression increased the occurrence of complications by 121 and 51%, respectively. High levels of PC have a protective effect against complications by 19%. Moreover, high levels of LVEF have a protective effect against complication by 17%.

## Discussion

This pioneering investigation demonstrates that PC moderates depression related to post-STEMI complications in in-hospital settings in a developing country. In other words, the results demonstrate that PC moderates post-STEMI in-hospital complications in relation to psychological symptoms (i.e. depression and anxiety) [3, 31]. Depression and PC were both independent predictors of these complications, affirming the findings of previous studies on the former predictor [4–6, 36]: PC militates against depression, while the latter predisposes patients to such complications, the most prolific of which was acute recurrent ischemia [3, 4, 7, 8, 31]. Approximately one-quarter (24%) of participants in this study developed some form of complication.

This study thus affirms previous investigations finding relatively high prevalence of in-hospital complications following STEMI, contrary to research that found no significant link between in-hospital complications and depression [37–41]. Numerous potential reasons for these divergent findings can be postulated, including differing operational definitions of depression itself, different data collection techniques (e.g. timing of depression evaluation), different sample sizes, and a lack of regard for depression moderators. Depression-measuring tools may have wide indications spanning numerous forms of illness, including subclinical CHD. In this study, HADS was used to measure depression, to differentiate depression among physically ill patients, which removes somatic indications that can obscure factors of heart disease but which may blunt depression detection [3, 6, 35]. As a result, this study is conservative in its estimation of the significance of the relationship between in-hospital complications and depression.

There are numerous possible causes of the link between complications and depression. For instance, physiologically, the experience of depression inhibits para-sympathetic neuronal activity, and activates sympathetic nervous activity, which gives a fillip to activities conducive to complications (e.g. inflammation, fibrillation threshold changes, decreased variability in heart rate, and increased aggregation of platelets) [2, 6, 8, 31]. Initial proliferation of such physiological variables immediately after AMI is highly linked to the development of complications [2, 6, 8, 31]. In terms of behavior, depression is linked to reduced enthusiasm, exercise, and healthy eating, and increased smoking and other unhealthy behaviors [3, 28, 31], albeit these usually play a minimal role in the experience of acute clinical/ health events [3, 28, 31].

Depression experienced during or in the wake of dangerous or critical events and experiences can be moderated by PC to reduce in-hospital complications, particularly in terms of improving coping skills [31, 42], and reducing anxiety, whose mechanisms are similar to depression [31, 42]. This entails that the association between in-hospital complications and anxiety is strong when PC is low, and vice-versa. Consequently, increased PC reduces in-hospital complications and creates a protective cardiac impact: it reduces anxiety and stimulation of the sympathetic nervous system, supporting parasympathetic nervous activity [31, 42], thus guarding from complications.

The empirical results of this study support general conclusions reached by previous researchers, and are of potential clinical importance with regard to depression being associated with lower LVEF [11]; every increase in LVEF units increases by 17% the protective effect against in-hospital complications [3, 28, 31]. Improved quality of life among STEMI patients is also linked to higher LVEF, thus enhancing the latter may improve the former, which may also control depression, high levels of which correspond to low prevalence of LVEF. The results of this investigation thus affirm the moderating role of PC on depression and improved LVEF.

## Conclusions

In patients with STEMI, depression is associated with increased risk of complications in the early phase. Perceived control has a protective and moderating effect on this relationship. Assessment of depression and enhancement of PC in this group might decrease complications, morbidity, and mortality.

### Limitations

The major limitation of this study was the exclusion of hemodynamically unstable patients, which might decrease the incidence of in-hospital complications. Moreover, data were collected from one major city in Jordan, which might limit the generalizability of the results.

## Data Availability

The datasets used and/or analyzed during the current study available from the corresponding author on reasonable request.

## Abbreviations

AMI: Acute myocardial infarction
CAS-R: Control attitude scale revised
CHD: Coronary heart disease
CVDs: Cardiovascular diseases
HADS: Hospital anxiety and depression scale
IRB: Institutional review board
LVEF: Left ventricular ejection fraction
PC: Perceived control
RA: Research assistant
STEMI: ST-segment elevation myocardial infarction
